# Radiogenomics Profiling for Glioblastoma-related Immune Cells Reveals CD49d Expression Correlation with MRI parameters and Prognosis

**DOI:** 10.1038/s41598-018-34242-9

**Published:** 2018-10-30

**Authors:** Hye Rim Cho, Hyejin Jeon, Chul-Kee Park, Sung-Hye Park, Seung Hong Choi

**Affiliations:** 1 Department of Radiology, Seoul National University Hospital, Seoul, Korea; 2 Center for Nanoparticle Research, Institute for Basic Science (IBS), Seoul, Korea; 3 Department of Neurosurgery, Seoul National University Hospital, Seoul, Korea; 4 Department of Pathology, Seoul National University Hospital, Seoul, Korea

## Abstract

Although there have been a plethora of radiogenomics studies related to glioblastoma (GBM), most of them only used genomic information from tumor cells. In this study, we used radiogenomics profiling to identify MRI-associated immune cell markers in GBM, which was also correlated with prognosis. Expression levels of immune cell markers were correlated with quantitative MRI parameters in a total of 60 GBM patients. Fourteen immune cell markers (i.e., CD11b, CD68, CSF1R, CD163, CD33, CD123, CD83, CD63, CD49d and CD117 for myeloid cells, and CD4, CD3e, CD25 and CD8 for lymphoid cells) were selected for RNA-level analysis using quantitative RT-PCR. For MRI analysis, quantitative MRI parameters from FLAIR, contrast-enhanced (CE) T1WI, dynamic susceptibility contrast perfusion MRI and diffusion-weighted images were used. In addition, PFS associated with interesting mRNA data was performed by Kaplan-Meier survival analysis. CD163, which marks tumor associated microglia/macrophages (TAMs), showed the highest expression level in GBM patients. CD68 (TAMs), CSF1R (TAMs), CD33 (myeloid-derived suppressor cell) and CD4 (helper T cell, regulatory T cell) levels were highly positively correlated with nCBV values, while CD3e (helper T cell, cytotoxic T cell) and CD49d showed a significantly negative correlation with apparent diffusion coefficient (ADC) values. Moreover, regardless of any other molecular characteristics, CD49d was revealed as one independent factor for PFS of GBM patients by Cox proportional-hazards regression analysis (*P* = 0.0002). CD49d expression level CD49d correlated with ADC can be considered as a candidate biomarker to predict progression of GBM patients.

## Introduction

The most aggressive form of brain cancer, called glioblastoma (GBM), is highly invasive and spreads rapidly, which makes it difficult to remove completely. GBM is also characterized by heterogeneity, angiogenesis, and strengthened cell proliferation. Although there have been many efforts of research and multi-modality treatment with surgical resection followed by chemotherapy and radiation therapy, patients average approximately 1 year of survival time^1^. Accordingly, targeting the tumor microenvironment (TME) of GBM is an emerging approach to overcome tumor resistance against the standard treatment^2–5^.

In the context of immune cells in TME, there are two major lineages of cells. One is the myeloid lineage, including macrophages, neutrophils, myeloid-derived suppressor cells (MDSC), dendritic cells (DC) and mast cells, and the other is the lymphoid lineage, including CD4+ helper T cells, regulatory T cells and CD8+ cytotoxic T cells, which have distinct functions during tumorigenesis^6–8^. Inter alia, macrophages and T cells present the most noticeable characteristics among the immune cells in TME. As noted in several studies^9–13^, there are two types of macrophages, M1 type and M2 type. The function of each type of macrophage around the tumor is significantly different. Pro-inflammation is the major characteristic of the M1 type, which is correlated with anti-tumorigenic functions in TME. In contrast, the main feature of the M2 type is anti-inflammation, which correlates with the pro-tumorigenic function in TME. Thus, they are known as tumor associated microglia/macrophages (TAMs), whose presence in tumors supports angiogenesis and invasion^9–13^. Helper T cells are also classified into two different types. Type 1 helper T cells (T_H_1) secrete pro-inflammatory cytokines and can be anti-tumorigenic; on the other hand, type 2 helper T cells (T_H_2) could be pro-tumorigenic by secreting anti-inflammatory cytokines, indicating that the ratio of T_H_1 to T_H_2 is crucial for tumor stage and grade^8,9,13,14^. As such, determining the various roles of TME immune cells has come to the light as a key point for diagnosis and prediction of cancer progression^8^.

CD system which was established in the 1^st^ International Workshop and Conference on Hyman leukocyte Differentiation Antigen (HLDA) is commonly used as cell markers in immunophenotyping^15,16^. It allows cells to be defined based on what molecules are present on their surface and to be often used to associate cells with certain immune functions regardless of HLA diversity of each patients^17^. Here, we chose fourteen CD markers based on the previous paper for identifying immune cells^8^.

Radiogenomics is the study linking medical images with the genomic profiles of human tumors. It has several benefits in terms of providing opportunities for non-invasive diagnostics and prognostics. For example, through radiogenomics, we could uncover imaging biomarkers that can identify the genomics of a disease, especially cancer, without using a biopsy^18^. Recently, although there have been a plethora of radiogenomics studies related to GBM, most of them used genomic information of tumor cells, not immune cells^19–22^. As 30-50% of the cells in gliomas are immune cells^23–27^, disclosing the information of immune cells in TME is important to understand fundamental details of the tumor-host interaction and predict the prognosis. Furthermore, provided that we are able to obtain immune cell information via the MRI findings, which is the general diagnostic method for brain tumors, it should contribute to both GBM diagnosis and treatment.

In this study, we used radiogenomics profiling to identify MRI-associated immune cell markers in GBM, which was also correlated with prognosis.

## Results

### Clinical characteristics of the GBM patients

The baseline epidemiologic and molecular characteristics are shown in Table 1. In brief, 35 male and 25 female patients with mean age of 54.22 ± 11.39 years at diagnosis of GBM were enrolled for this study. GBMs were predominantly located at the supratentorial brain (97%, *n* = 59), including frontal lobe (55%, *n* = 33), parietal lobe (35%, *n* = 21), temporal lobe (57%, *n* = 34), occipital lobe (7%, *n* = 4), insula (13%, *n* = 8), deep gray matter (23%, *n* = 14), corpus callosum (13%, *n* = 8) and mid-brain (8%, *n* = 5). In molecular characteristics, nine (15%) and two (3%) patients (15%) presented IDH1- and IDH2-mutated tumors, respectively. Half of the patients (*n* = 30) had MGMT promoter methylation, and 13 patients showed ATRX mutation, while none of patients had 1p/19q co-deleted tumor.

**Table 1:** Characteristics of 60 patients with glioblastoma multiforme.

Characteristics	N
Age	54.22 ± 11.39*
Sex	
Male	35
Female	25
Tumor location**	
Supratentorial	59
Frontal	33 (24)
Parietal	21 (18)
Temporal	34 (22)
Occipital	4 (4)
Insula	8 (7)
Deep gray matter	14 (14)
Corpus callosum	8 (8)
Mid-brain	5
Infratentorial	2
Infratentorial	2 (1)
Histopathologic assay	
IDH1	
mutant	9
wildtype	51
IDH2	
mutant	2
wildtype	58
1p/19q	
co-deleted	0
non co-deleted	60
MGMT promoter	
methylated	30
unmethylated	30
ATRX	
mutant	13
wildtype	47

*Mean value ± standard deviation.

**The tumor locations were classified according to the tumor epicenter.

Parentheses: the number of patients with tumors involving more than one location.

### Immune cell RNA expression level in GBM

**Figure 1:**
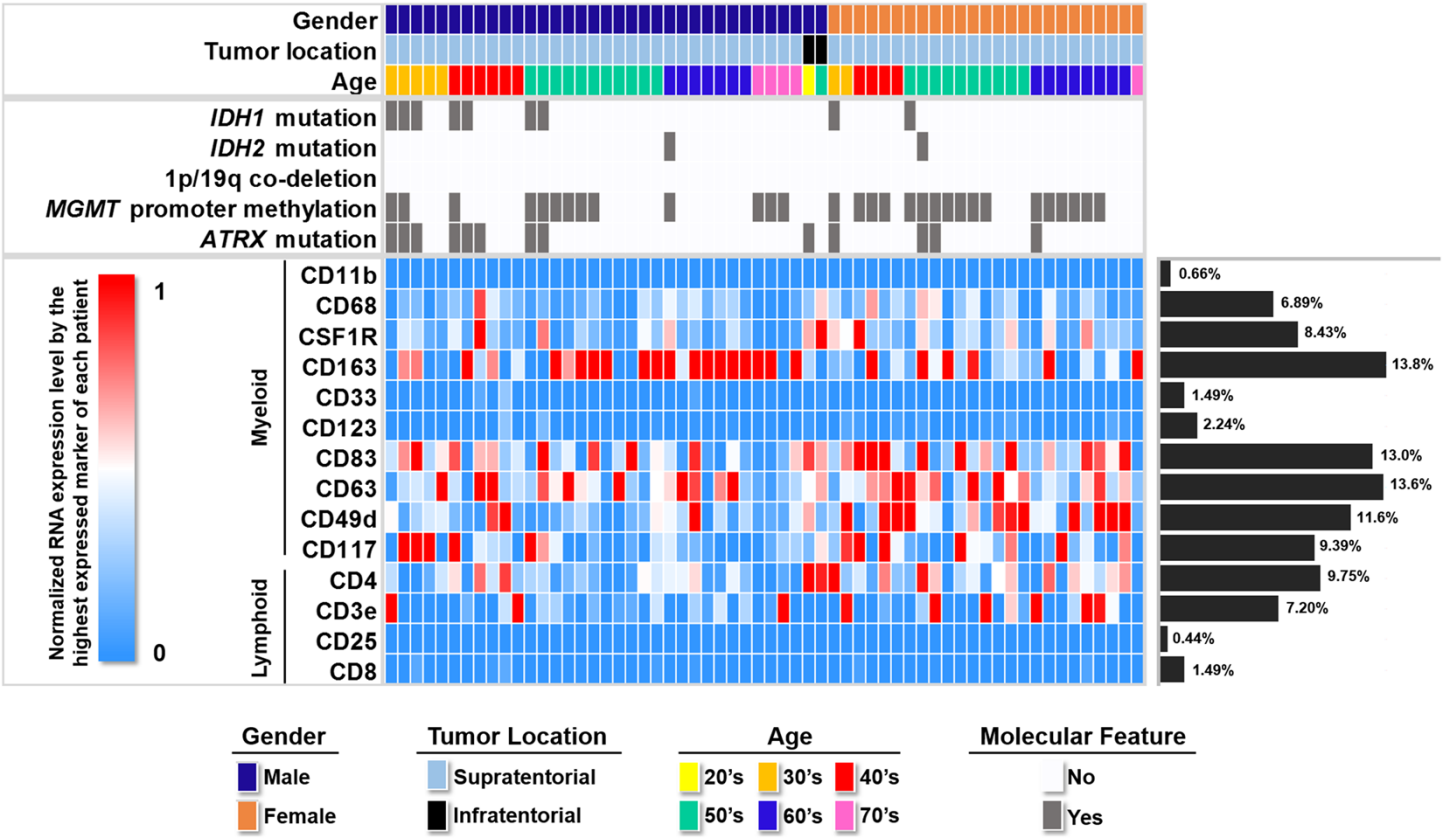
Illustration of age, gender, tumor location, genetic information and normalized RNA expression level of immune cell markers in each patient.

Normalized expression levels of CD11b, CD68, CSF1R, CD163, CD33, CD123, CD83, CD63, CD49d, CD117, CD4, CD3e, CD25 and CD8 in each patient were summarized in Fig. 1. We found that myeloid lineage immune cell markers were more dominant than lymphoid lineage markers. Among the myeloid lineage immune cell markers, CD163, CD63, CD83 and CD49d showed RNA expression levels of more than 10%.

### TAM and T cell markers correlated with nCBV, ADC, volume and necrosis values

**Figure 2:**
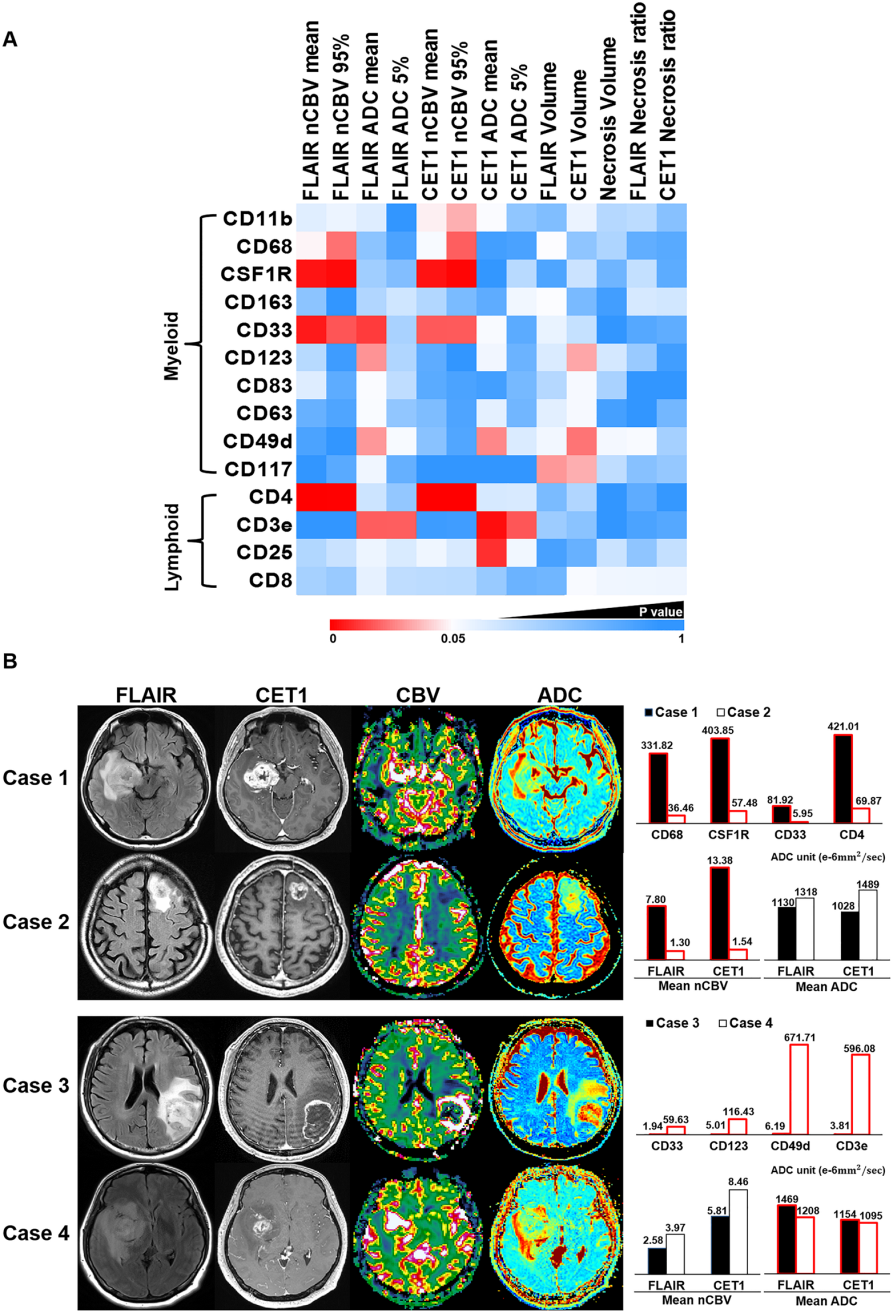
Illustration of correlation between MRI parameters and immune cell markers (**A**), and representative cases (**B**). (**A**) Case 1 and Case 2 show high and low expression level of CD68, CSF1R, CD33 and CD4, respectively, which have significant correlation with nCBV values. In Case 3 and Case 4, high and low expression levels of CD33, CD123, CD49d and CD3e are significantly correlated with ADC values.

We performed Pearson’s correlation analysis to determine the association between immune cell markers and MRI parameters, and Fig. 2A, Supplementary Fig. 1 and Table 2 summarize the correlation. The expression levels of CD68, CSF1R, CD33 and CD4 showed significant positive correlations with nCBV values based on both region of interests (ROIs) from FLAIR and CE T1WI and that of CD11b had a significant positive correlation with nCBV values only from CE T1WI (Fig. 2B; Case 1 and 2). We found significant negative correlations between the expression levels of CD49d and CD3e and ADC values from both FLAIR and CE T1WI, and those of CD33 and CD123, and CD25 were negatively correlated with ADC values from FLAIR and CE T1WI, respectively (Fig. 2B; Case 3 and 4). Tumor volumes based on FLAIR or CE T1WI had significant negative correlations with the expression levels of CD123, CD49d and CD117, but no immune cell markers showed a significant correlation with tumor necrosis or necrosis ratio.

**Table 2:** Correlation analysis between immune cell markers and MRI values.

		FLAIR nCBV mean	FLAIR nCBV 95%	FLAIR ADC mean	FLAIR ADC 5%	CET1 nCBV mean	CET1 nCBV 95%	CET1 ADC mean	CET1 ADC 5%	FLAIR Volume	CET1 Volume	Necrosis Volume	FLAIR Necrosis Ratio	CET1 Necrosis Ratio
CD11b	*r*	0.179	0.203	−0.176	0	0.255	0.271	−0.243	−0.088	−0.074	−0.199	−0.122	−0.13	−0.08
	*P*	0.1702	0.1205	0.1787	0.9994	0.0494	0.0363	0.0616	0.5054	0.5747	0.127	0.3534	0.3207	0.5439
CD68	*r*	0.254	0.292	−0.084	0.035	0.232	0.301	−0.028	0.033	0.248	0.087	0.115	−0.05	0.044
	*P*	0.0505	0.0236	0.5249	0.7926	0.0746	0.0196	0.8311	0.8026	0.0556	0.5097	0.38	0.7062	0.7407
CSF1R	*r*	0.364	0.385	−0.101	0.075	0.366	0.404	−0.017	0.125	−0.036	−0.148	−0.061	−0.138	−0.046
	*P*	0.0043	0.0024	0.4408	0.5678	0.0041	0.0014	0.8956	0.3406	0.7829	0.2604	0.6444	0.2917	0.7246
CD163	*r*	−0.084	−0.002	0.118	0.154	0.119	0.069	0.048	0.214	0.245	0.068	−0.027	−0.163	−0.156
	*P*	0.5231	0.9879	0.3704	0.2411	0.3653	0.5984	0.7177	0.1002	0.0589	0.6039	0.8361	0.213	0.2326
CD33	*r*	0.354	0.305	−0.321	−0.11	0.303	0.302	−0.233	−0.046	−0.197	−0.141	−0.015	0.041	0.049
	*P*	0.0056	0.0178	0.0125	0.403	0.0186	0.0189	0.0726	0.7284	0.1322	0.2839	0.9112	0.7549	0.7112
CD123	*r*	0.125	0.025	−0.279	−0.117	0.047	0.015	−0.213	−0.057	−0.169	−0.273	−0.161	−0.095	−0.027
	*P*	0.3416	0.8514	0.0306	0.3713	0.7189	0.9084	0.1022	0.6642	0.1981	0.0345	0.2205	0.468	0.8393
CD83	*r*	0.175	0.047	−0.241	−0.133	0.045	0.034	−0.028	0.066	−0.122	−0.229	−0.11	0.014	−0.005
	*P*	0.1814	0.7223	0.064	0.3098	0.7323	0.7952	0.8293	0.6157	0.354	0.0781	0.4025	0.9154	0.9689
CD63	*r*	0.054	0.037	−0.24	−0.089	0.073	0.036	−0.187	−0.062	−0.182	−0.229	−0.03	0.014	0.064
	*P*	0.6794	0.7787	0.0646	0.5012	0.5813	0.7837	0.1536	0.6384	0.1642	0.0782	0.8218	0.914	0.6273
CD49d	*r*	0.034	−0.003	−0.278	−0.233	0.081	0.038	−0.284	−0.169	−0.217	−0.291	−0.223	−0.234	−0.106
	*P*	0.7971	0.9821	0.0314	0.0736	0.5373	0.7713	0.0281	0.1974	0.0961	0.0242	0.087	0.0718	0.4184
CD117	*r*	−0.007	−0.044	−0.212	−0.053	−0.015	0.014	−0.001	0.012	−0.278	−0.271	−0.133	−0.079	−0.092
	*P*	0.9554	0.7397	0.104	0.6859	0.9118	0.9138	0.9961	0.9245	0.0313	0.036	0.311	0.5461	0.4859
CD4	*r*	0.447	0.431	−0.149	−0.09	0.433	0.433	−0.163	−0.166	−0.069	−0.118	−0.055	−0.048	0.018
	*P*	0.0003	0.0006	0.2551	0.4934	0.0005	0.0005	0.2137	0.2049	0.5986	0.37	0.6789	0.7167	0.8896
CD3e	*r*	−0.005	−0.015	−0.301	−0.3	0.024	0.026	−0.376	−0.304	−0.099	−0.082	−0.016	−0.034	−0.041
	*P*	0.9686	0.9085	0.0195	0.0197	0.8552	0.8419	0.0031	0.0181	0.4511	0.5324	0.9023	0.7968	0.7567
CD25	*r*	0.118	0.145	−0.204	−0.168	0.209	0.166	−0.33	−0.215	−0.031	−0.055	−0.146	−0.087	−0.139
	*P*	0.3677	0.2697	0.1179	0.1991	0.1098	0.2051	0.01	0.0985	0.8113	0.6787	0.2644	0.5101	0.2896
CD8	*r*	0.107	0.095	−0.191	−0.133	0.13	0.128	−0.094	0.058	−0.061	−0.233	−0.213	−0.21	−0.207
	*P*	0.4167	0.4696	0.1445	0.3105	0.3206	0.3311	0.4751	0.6605	0.6418	0.0727	0.1027	0.1075	0.1134

*r*: Pearson correlation coefficient.

### Immune cell makers correlated with PFS

**Figure 3:**
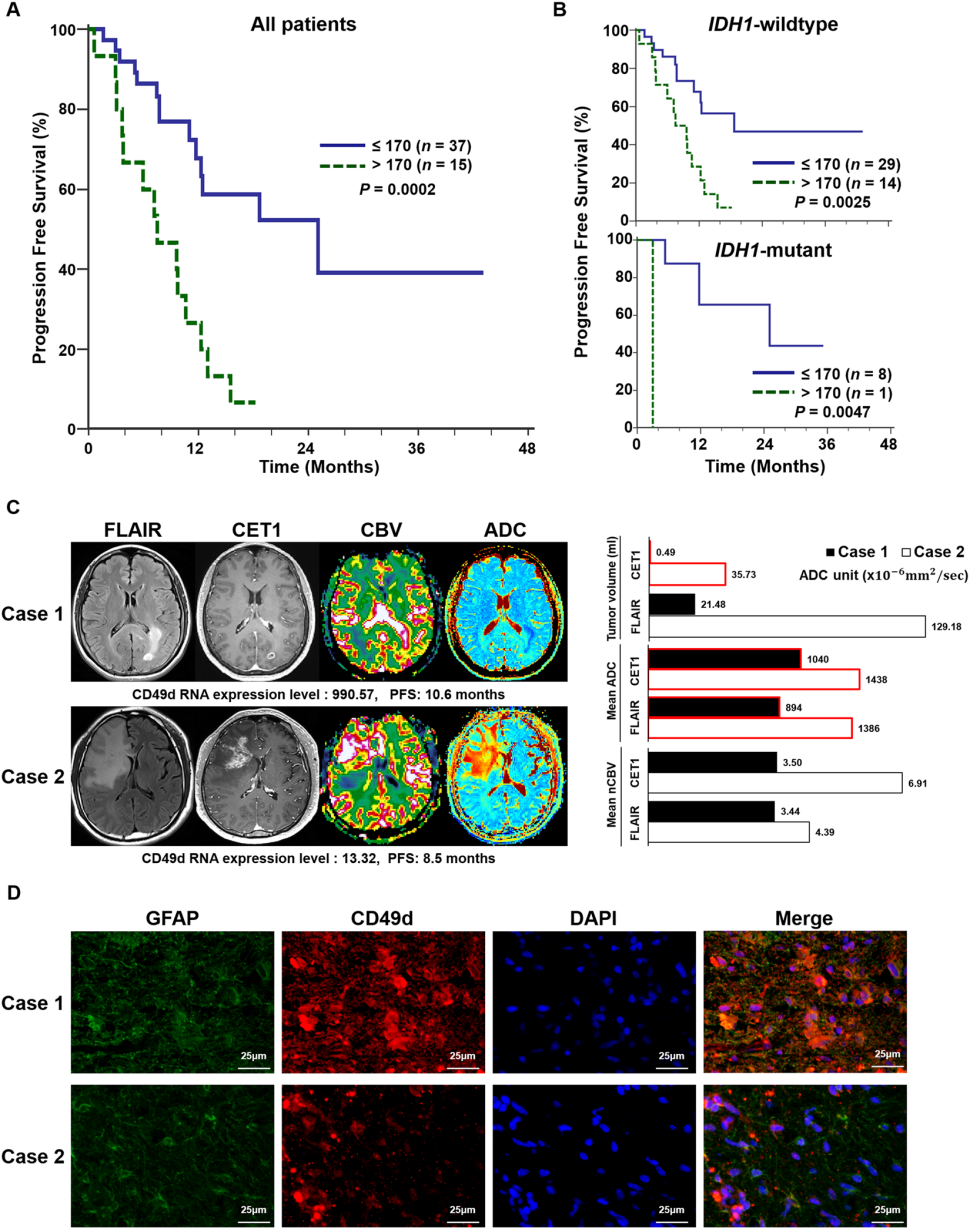
Kaplan-Meier estimates of PFS according to the CD49d expression level (**A** and **B**) and representative cases (**C**). PFS of GBM patients with low expression level of CD49d (≤170) was significantly longer than that of patients with high expression level of CD49d (>170) (**A**), which was independent of IDH1 mutation status (**B**). In all patients, there was a significant difference in PFS between low and high CD49d expression tumors (median, 25.1 [95% CI, 12.3-25.1] vs 7.5 [95% CI, 3.5-10.6] months; *P* = 0.0002, log-rank test). Patients with IDH1-wildtype GBMs also had a significant difference in PFS between low and high CD49d expression tumors (median, 18.7 [95% CI, 11.0-18.7] vs 7.5 [95% CI, 3.5-12.3] months; *P* = 0.0025, log-rank test). In patients with IDH1-mutant GBMs, there was also a significant difference in PFS between low and high CD49d expression tumors (median, 25.1 [95% CI, 11.7-25.1] vs 3 months; *P* = 0.0047, log-rank test). (**C**) Case 1 and Case 2 show high and low expression level of CD49d, respectively, which is negatively correlated with ADC mean values based on both FLARI and CE T1WI and tumor volume on CE T1WI. (**D**) Immunohistochemistry results for CD49d are correlated with CD49d RNA expression level. (Glial fibrillary acidic protein: GFP, CD49d: RFP, nucleus: DAPI).

PFS was correlated with fourteen immune cell markers, and eight makers showed statistical significance (*P* < 0.05), including myeloid cell markers (e.g., CD11b, CD123, CD33, CD163, CD63 and CD49d) and lymphoid cell marker genes (e.g., CD25 and CD8). Each gene expression level of higher threshold was associated with poor PFS (Fig. 3, Supplementary Fig. 2, and Supplementary Tables 1-3). Then, we executed Cox proportional hazards model analysis, including eight genes significantly associated with PFS and previously reported prognostic factors (e.g., IDH1, IDH2, 1p/19q, MGMT and ATRX status) (Table 3), which revealed that only CD49d was significant among thirteen covariates (Table 2, *P* = 0.0007). In all patients, there was a significant difference in PFS between low and high CD49d expression tumors (median, 25.1 [95% CI, 12.3-25.1] vs 7.5 [95% CI, 3.5-10.6] months; *P* = 0.0002, log-rank test), which was independent of IDH1 mutation status (Fig. 3 and Supplementary Tables 2 and 3).

**Table 3:** Results of Cox proportional hazards model analysis.

Covariate	b	SE	*P*	Exp (b)	95% CI of Exp (b)
CD49d expression level (≥170)	1.3476	0.3954	0.0007	3.848	1.7728 to 8.3525

b - coefficient estimates; 95% CI - 95% confidence interval.

Exp(b) - hazard ratio value; SE - standard error of coefficient estimates b.

## Discussion

In this study, we applied radiogenomics profiling to the association between quantitative MRI features and expression levels of immune cell markers in GBM patients, which was also correlated with PFS. We found that myeloid lineage immune cell markers (e.g., CD163, CD63, CD83 and CD49d) were more dominant than lymphoid lineage markers in GBMs. In terms of CBV values based on FLAIR or CE T1WI, the expression levels of CD68, CSF1R, CD33, CD4 and CD11b showed positive correlations. In addition, the expression levels of CD49d, CD3e, CD33, CD123 and CD25 were found to have negative correlations with ADC values based on FLAIR or CE T1WI. The expression levels of CD123, CD49d and CD117 had negative correlations with tumor volumes. PFS was also correlated with myeloid cell markers (e.g., CD11b, CD123, CD33, CD163, CD63 and CD49d) and lymphoid cell marker genes (e.g., CD25 and CD8). Among them, CD49d was the most important prognostic marker regardless of genetic status of GBM.

TAMs are known to have a role in tumor angiogenesis; therefore, we assumed that TAMs can affect the increase in nCBV values, which can be simultaneously correlated with TAM markers (e.g., CD11b, CD68 and CSF1R). Recent studies have elaborated an important role of CSF1R-colony stimulating factor-1 receptor in the brain TAM; for example, inhibition of CSF1R either reduces^28^ or depolarizes TAMs^29^. In addition, CD33, a MDSC marker, has high correlation with nCBV values. The suppressive activity of MDSC is one of the most prevalent mechanisms of immune evasion in patients^30^, which occurs as a consequence of the aberrant myelopoiesis that arises in cancer. MDSCs are mobilized during tumorigenesis and infiltrate developing tumors, where they promote tumor angiogenesis^31^ and disrupt major immunosurveillance mechanisms. Among the lymphoid lineage markers, CD4 is significantly positively correlated with nCBV values. As we described above, nCBV seem to be related to the function of TAMs, which induce angiogenesis in tumors. Regulatory T cell and type 2 helper T cells (T_H_2) marked by CD4 express Interleukin-10^32^, Interleukin-4 and Interleukin-13^33^, which are cytokines for M2-type macrophage polarization^11,34,35^. In other words, TAMs can be induced primarily by pro-tumorigenic roles through suppressive immunosurveillance and anti-inflammatory cytokines, which can cause increase in nCBV resulting from angiogenesis, one of the key roles of TAMs in GBM^10,23^.

CD49d, CD33, CD123, CD3e, and CD25, which mark bone marrow-derived cells (MDSCs) dendritic cells (DC), T helper cells, cytotoxic T cells and regulatory T cells, respectively, are negatively correlated with ADC values. Dendritic cells are known as antigen presenting cells derived from the bone marrow, which induces innate and adaptive immune responses^36^. When tumors occur, immune cells, including MDSCs, which are the precursor of DCs, macrophages and granulocytes^9,12,13^, DCs and T cells, are recruited from bone marrow and converge on the tumor side by the immune system^13^. We believe that tumor cellularity, measured by ADC value, can be affected by immune cell infiltration as well as tumor tissue itself. In addition, myeloid cells seem to play a more important role than lymphoid cells in tumor cellularity.

In addition, CD123, CD117, and CD49d, which mark DCs, mast cells and bone marrow-derived cells^8^, respectively, are negatively correlated with tumor volume. Before they are recruited by tumors, DCs and mast cells induce innate and adaptive immune responses to regress tumors and prevent relapse^9^. CD49d was revealed as a marker of hematopoietic bone marrow-derived cells^37^. DCs and mast cells activate T cells to remove tumors^8,12^, which can be explained by the finding that these immune cells recruit in the early stage of gliomagenesis.

In terms of PFS, as the previous study has been reported that high CD49d expression is associated with poor survival in chronic lymphocytic leukemia^38^, it is remarkable that CD49d was discovered an independent biomarker regardless of any other molecular characteristics, even though TAM markers are prominent among the immune cell population and correlate with several imaging features. CD49d is also known as integrin α4, forming a heterodimer with integrin β1 (VLA4), which is the specific marker of hematopoietic cells, namely, bone marrow-derived cells. Bowman RL *et al*. demonstrated that microglia specifically repress integrin α4 (CD49d), enabling its utility as a discriminatory marker between microglia and bone marrow-derived macrophages in primary and metastatic disease in both mouse and human^37^. Several studies reported that the brain-resident microglia and the infiltrating monocytes/macrophages of blood are the major glioma-associated inflammatory cells that constitute the tumor microenvironment^39,40^. Particularly, a recent report^41^ and a clinical study^42^ revealed that monocytes/macrophages, but not microglia and lymphocytes, are the most predominant TAMs in GBM. Monocytes, cells of the myeloid lineage, are released during inflammation and differentiate into macrophages to maintain immune homeostasis^43^. A previous study suggested that circulating monocytes are cytotoxic to tumor cells^44^; however, when monocytes reach the tumor mass, the tumor molecular milieu induces differentiation to new cell types per tumor requirement^45^. A recent report indicated that reduction in the tumor-promoting effects of monocytes/macrophages in GBM can be considered as an adjuvant treatment for glioma^46^. However, the fate of the GBM-adhered monocytes/macrophages and their effect on GBM growth are still obscure. Adhesion molecules are known to mediate cell–cell interactions, particularly between immune cells and target tissues. Vascular cell adhesion molecule-1 (VCAM-1) is an inducible adhesion molecule that facilitates tight attachment to the monocyte/macrophage-associated integrin α4β1 (VLA4)^47^. VCAM-1 expressed on the surface of tumors interacts with VLA4 on monocytes/macrophages, which promotes tumor invasion, angiogenesis and metastasis^10^. In short, higher CD49d expression level in GBM is thought to induce TAMs to adhere to GBM, increasing cellularity and resulting in a decrease in ADC on MRI, attenuated tumor aggressiveness and poorer progression of patients. The previous studies have been reported that MRI parameters (e.g., nCBV and ADC) are used for evaluation of GBM patients’ overall survival (OS) and PFS and they are related to poor progression of GBM. For instance, high nCBV and low ADC values are results of poor PFS^48^. Therefore, our findings related to immune cell markers and MR imaging parameters can follow antecedent researches.

There are some limitations in this study in addition to the retrospective design. First, in this study, we did not perform experiments providing direct evidence to show the relationship between immune cell migration, angiogenesis and cellularity. Thus, future study is warranted. Second, we could not see the interactions between immune and cancer cells, which need further *in vitro* and *in vivo* experiments to be translated to future clinical studies.

Our radiogenomics profiling reveals that immune cell markers such as TAM markers have significant correlations with nCBV and ADC values, and CD49d expression level correlated with ADC can be considered as a candidate biomarker to predict progression of GBM patients. We believe that our results can be used for understanding the GBM microenviroment and development of evaluation and treatment strategies.

## Methods

This retrospective human study was approved by the institutional review board of Seoul National University Hospital, which waived the requirement of obtaining informed consent.

### Patient population

Between August 2012 and December 2015, 258 patients who were initially diagnosed with GBM at our institution were consecutively recruited. The inclusion criteria were as follows: the patient (a) had a histopathologic diagnosis of GBM without other cell components based on the World Health Organization 2016 criteria; (b) underwent conventional, diffusion-weighted imaging (DWI) and DSC perfusion MR imaging 24-48 hours before surgery; (c) had available tumor samples in the brain tumor bank of our institute; and (d) underwent the standard treatment of near-total resection, concomitant chemoradiotherapy (CCRT) and adjuvant temozolomide medication. As a result of these inclusion criteria, 60 patients were included in our study. All tumor samples used in this study were snap-frozen in liquid nitrogen as soon as possible during the surgery and stored at −80 °C.

### RNA isolation and real-time PCR

The total RNA of each tissue sample was isolated using the QIAquick RNeasy Mini kit (Qiagen) according to the manufacturer’s instructions, and the quality of the RNA was verified by an Agilent 2100 Bioanalyzer (Agilent Technologies). Reverse transcription was performed with RevertAid H Minus Reverse Transcriptase (Thermo). Briefly, reverse transcription was carried out in a volume of 100 μl with 2.0 μg RNA, 15 pmol of oligo deoxythymidine primer, 20 µl of 5Χ RT Buffer, and 20 μl each of 2.5 mM dNTP mix, RNase inhibitor, and reverse transcriptase. RT conditions were as follows: 10 minutes at 65 °C, 60 minutes at 42 °C, 10 minutes at 25 °C, and 10 minutes at 70 °C.

Real-time PCR was performed in a Rotor-Genes Q cycler machine (Qiagen) using Rotor-Genes SYBR Green PCR kit (Qiagen) according to the manufacturer’s instructions in a total volume of 20 µl. Cycling conditions for the immune cell markers and GAPDH were 10 minutes at 95 °C, 40 cycles of 10 seconds at 95 °C, 15 seconds at optimal Tm, and 20 seconds at 72 °C. The sequences of the primers were as follows:

CD11b; 5′-caactatggagaatggtcctaagct-3′/5′-tgtccagtcgctctcttctcttc-3′, CSF1R; 5′-tttggggctagacagactgg-3′/5′-cctgagctgagtgtggtctg-3′, CD123; 5′-gggggtctgcctcaatct-3′/5′-caccacccgttaggaatgtc-3′, CD33; 5′-tttaacaccccacaggcaat-3′/5′-gcacagatttgattccacga-3′, CD3e; 5′-tccctaccaaccccctaatc-3′/5′-tacggagatgcaaatgacca-3′, CD25; 5′-agttttcagcagggtccaga-3′/5′-ggggagagtgcacagatgag-3′, CD8; 5′-ctggcctctgctcaactagc-3′/5′-gaagtgcatgtttgggacag -3′, CD68; 5′-aaagtttctcctgccccagt -3′/5′-gcagaaagcaataagcacca-3′, CD163; 5′-tgagccacactgaaaaggaa-3′/5′-gctccattcaatagtccaggtc-3′, CD83; 5′-caggtccacggtctgttctt-3′/5′-cttcgtgaagtcccttctgc-3′, CD63; 5′-tttgtcgaggttttgggaat-3′/5′-cagatgaggaggctgaggag-3′, CD49d; 5′-taccaagaatgcgtttgcag-3′/5′-gagcattcaacttcccttgg-3′, CD117; 5′-ccagaagcttccatagtggtg-3′/5′-agtgccttaagtgcaggtgaa-3′, CD4; 5′-ggctctcaccagtggctagt-3′/5′-ccttcatccctgctcgtaaa-3′ and GAPDH; 5′-ggcattgctctcaatgacaa-3′/5′-atgtaggccatgaggtccac-3′. A standard curve was generated and a nontemplate control was run with every assay to correlate the threshold (Ct) values from the amplification plots to copy number. All samples were run in duplicate, and the average value was used.

We normalized immune cell markers by the highest expressed marker in each patient.

### MRI protocol

All patients underwent conventional, DWI and DSC perfusion MRI using a 3 T scanner (Verio; Siemens Healthcare Sector) with a 32-channel head coil. The conventional MRI included T1-weighted imaging (T1WI), such as transverse spin-echo imaging, before and after contrast enhancement or multi-planar reconstructed transverse, coronal imaging with a sagittal three-dimensional magnetization prepared rapid acquisition gradient echo (3D-MPRAGE) sequence before and after contrast enhancement, and transverse T2-weighted imaging (T2WI) with turbo spin-echo sequences and FLAIR images. Contrast-enhanced (CE) T1WI was acquired after the intravenous administration of gadobutrol (Gadovist®, Bayer Schering Pharma) at a concentration of 0.1 mmol per kilogram (mmol/kg) of body weight. The transverse spin-echo T1-weighted imaging was obtained with the following parameters: repetition time (TR), 558 ms; echo time (TE), 9.8 ms; flip angle (FA), 70°; matrix, 384 × 187; field-of-view (FOV), 175 × 220 mm; section thickness, 5 mm; and number of excitations (NEX), 1. We obtained the 3D-MPRAGE sequences using the following parameters: TR, 1500 ms; TE, 1.9 ms; FA, 9°; matrix, 256 × 232; FOV, 220 × 250; section thickness, 1 mm; and NEX, 1. The parameters of the transverse T2-weighted imaging were as follows: TR, 5160 ms; TE, 91 ms; FA, 124-130°; matrix, 640 × 510–580; FOV, 175-199 × 220; section thickness, 5 mm; and NEX, 3. The parameters for transverse FLAIR were a TR of 9000 ms, a TE of 97 ms, a TI of 2500 ms, an FA of 130°, a matrix of 384 × 348, an FOV of 199 × 220, a section thickness of 5 mm and an NEX of 1.

DWI was performed with a single-shot, spin-echo, echo-planar imaging (EPI) sequence in the axial plane before the injection of contrast material with b-values of 0 and 1000 sec/mm^2^, a TR of 6300 ms, a TE of 92 ms, an FA of 180°, a matrix of 240 × 240, an FOV of 240 × 240, a section thickness of 3 mm and an NEX of 3. DWI was acquired in three orthogonal directions and combined into a trace image. ADC maps were calculated on a voxel-by-voxel basis with the software that was incorporated into the MRI unit using these data.

The transverse DSC perfusion MRI was obtained with single-shot, gradient-echo, echo-planar sequences during the intravenous administration of gadobutrol at a concentration of 0.1 mmol/kg of body weight at a rate of 4 ml/sec using a power injector (Spectris; Medrad). A 30-ml bolus injection of saline followed at the same injection rate. For each section, 60 images were acquired at intervals equal to the TR. The parameters were as follows: TR, 1500 ms; TE, 30 ms; FA, 90°; matrix, 128 × 128; section thickness, 5 mm; intersection gap, 1 mm; FOV, 240 × 240 mm; sections, 15-20; voxel size, 1.875 × 1.875 × 5 mm^3^; pixel bandwidth, 1563 Hz; and total acquisition time, 1 minute 30 seconds.

### Image post-processing and data analysis

The conventional MR images, ADC maps, and DSC PWI were digitally transferred from the picture archiving and communication system workstation to a personal computer for further analysis. The relative CBV (rCBV) was obtained with a dedicated software package (nordicICE; Nordic Imaging Lab, Bergen, Norway) that applied an established tracer kinetic model to the first-pass data^49,50^. First, realignment was performed to minimize patient motion during the dynamic scans. A gamma-variate function, which approximates the first-pass response as it would appear in the absence of recirculation, was used to fit the 1/T2* curves to reduce the effects of recirculation. To reduce the contrast agent leakage effects, the dynamic curves were mathematically corrected by using leakage correction package available on the dedicated software^51^. After the elimination of recirculation and leakage of the contrast agent, rCBV was computed with numeric integration of the curve. To minimize variances in rCBV in an individual patient, the pixel-based rCBV maps were normalized by dividing every rCBV value in a specific section by the rCBV value in the unaffected white matter, and finally normalized rCBV (nCBV) maps were generated^52^.

Using a dedicated software package (nordicICE), co-registrations between the structural images (e.g., FLAIR images and CE T1WI) and the nCBV and ADC maps were performed based on geometric information stored in the respective data sets. The differences in the slice thickness between images were corrected automatically by re-slicing and co-registration based on the underlying structural images. The nCBV and ADC maps were displayed as color overlays on the both FLAIR images and CE T1WI.

One neuroradiologist (S.H.C. with 16 years of brain MR imaging experience) who was blinded to the clinical data drew polygonal ROIs that contained the entire enhancing lesions in each section of the co-registered images. Areas of necrosis, hemorrhage, or non-tumor macro-vessels that were evident on the CE T1WI were excluded from the ROIs. Then, the ROIs of T2 high SI lesions, regardless of contrast enhancement, were also defined on each transverse FLAIR image, avoiding the cystic and necrotic regions and the macrovessels. Because the ROI placement was conducted on the nCBV and ADC map co-registered with structural images, the margin of the lesions could be defined with confidence. The entire volume of contrast-enhancing lesions, T2 high SI lesions, and necrosis, which was defined as a hypointense area without contrast enhancement on CE T1WI within the mass on the FLAIR images, was calculated.

The data acquired from each section were summed to derive the voxel-by-voxel ADCs and nCBVs for the entire tumor extent based on both CE T1WI and FLAIR images by using nordic ICE. The ADC and nCBV histograms were plotted with ADC and nCBV on the respective x-axis with a bin size of 3 × 10^−5^ mm^2^/sec and 0.1, respectively, whereas the y-axis was expressed as a percentage of the total lesion volume by dividing the frequency in each bin by the total number of analyzed voxels. For further quantitative analysis, the cumulative number of observations in all bins up to the specified bin was mapped on the y-axis as a percentage in the cumulative histograms. The 5th percentile point for ADC (5% ADC) and 95th percentile point for nCBV (95% nCBV) were derived (the Xth percentile point is the point at which X% of the voxel values that form the histogram are found to the left of the histogram)^53,54^.

### Immunohistochemistry staining

Antibodies for IHC analysis included mouse anti-human Integrin alpha4 (CD49d) antibody (sc-365209, Santa Cruz Biotechnology, Inc.) and rabbit anti-human Glial fibrillary acidic protein (GFAP) antibody (ab7260, abcam). Paraffin sections (4 μm) were dewaxed and rehydrated, Antigen retrieval was performed in a microwave by placing the sections in epitope retrieval solution (0.01 M citrate buffer, pH 6.0) for 20 minutes, then incubated in 3% hydrogen peroxide for 20 min at room temperature. The paraffin sections were then blocked with 3% BSA for 30 min, stained with antibodies for 1 hour at room temperature, washed with wash buffer (S3006, Dako) and stained with secondary antibody (A-11008, Alexa Fluor 488 by Invitrogen^TM^; A-11032, Alexa Fluor 594 by Invitrogen^TM^) for 30 min at room temperature. DAPI was used to stain the nucleus.

### Statistical Analysis

All statistical analyses were performed using two commercial software programs (MedCalc version 17.2, MedCalc Software). A *P* value < 0.05 was considered statistically significant. Kolmogorov-Smirnov’s test was used to determine whether the variables followed normal distribution. Non-parametric data are presented as median and interquartile range (IQR, range from the 25th to the 75th percentile), and parametric data are shown as the mean ± standard deviation. Pearson’s correlation analysis for parametric data was performed for the correlation between the expression level of immune cell markers and quantitative imaging parameters.

The progression-free survival (PFS) was assessed using the Kaplan-Meier method according to the expression level of immune cell markers, which were compared using log-rank tests. GBM progression was defined according to RANO criteria^55^. We only recorded the first progression. PFS was calculated from the date of surgery to that of GBM progression, death, the last confirmation of no evidence of disease, or the most recent follow-up examination. Patients without an event were censored at the date of the most recent follow-up, regardless of whether they were scheduled for future follow-up or they had been lost to follow-up. Eight patients who expired from progression-unrelated conditions (e.g., infarction and infection) were excluded from PFS analysis. To determine thresholds in each immune cell marker expression level for PFS, receiver operating curve analysis was used. Multivariate analysis was performed using the Cox proportional hazards model, which was adjusted for the prognostic factors including the expression level of immune cell markers and IDH1 mutation status.

## Electronic supplementary material

Supplementary Information
