# Supplementary material for: Radiogenomics Profiling for Glioblastoma-related Immune Cells Reveals CD49d Expression Correlation with MRI parameters and Prognosis
Source: Sci Rep. 2018 Oct 30;8:16022. doi: 10.1038/s41598-018-34242-9 (PMC6207678; doi:10.1038/s41598-018-34242-9)
Supplement: Supplementary file 1 — Supplementary Information [file 41598_2018_34242_MOESM1_ESM.doc]

Supplementary Materials for

**Radiogenomics Profiling for Glioblastoma-related Immune Cells Reveals CD49d Expression Correlation with MRI parameters and Prognosis**

Hye Rim Cho^1,2#^, Hyejin Jeon^1,2#^, Chul-Kee Park^3^, Sung-Hye Park^4^, Seung Hong Choi^1,2*^

^1^Department of Radiology, Seoul National University Hospital, Seoul, Korea

^2^Center for Nanoparticle Research, Institute for Basic Science (IBS), Seoul, Korea

^3^Department of Neurosurgery, Seoul National University Hospital, Seoul, Korea

^4^Department of Pathology, Seoul National University Hospital, Seoul, Korea

^#^ Hye Rim Cho and Hyejin Jeon contributed equally to this study

**Supplementary Figures**

**
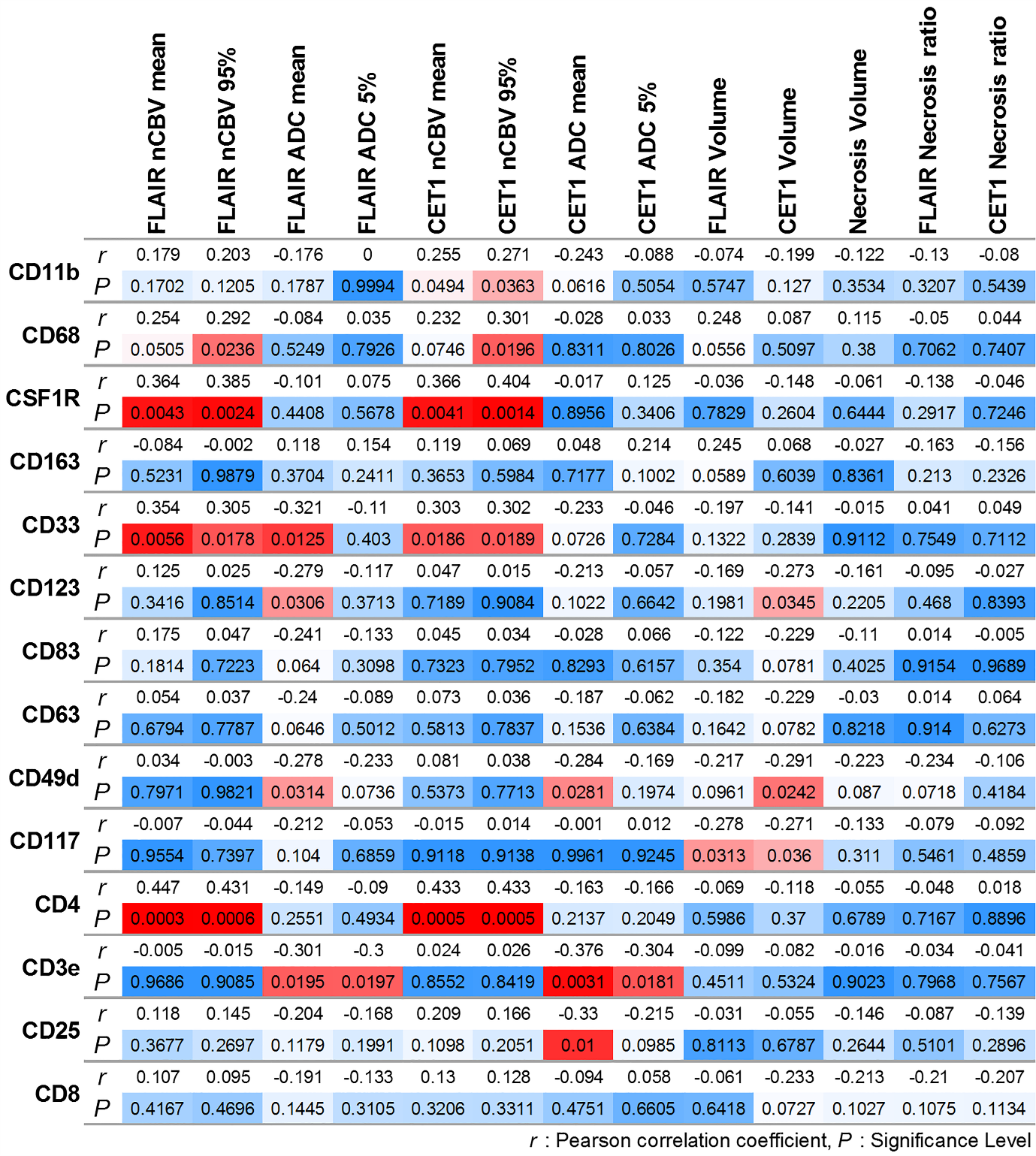
**

**Supplementary Figure 1. Correlation analysis between immune cell markers and MRI values, in which *P* values are labeled.**


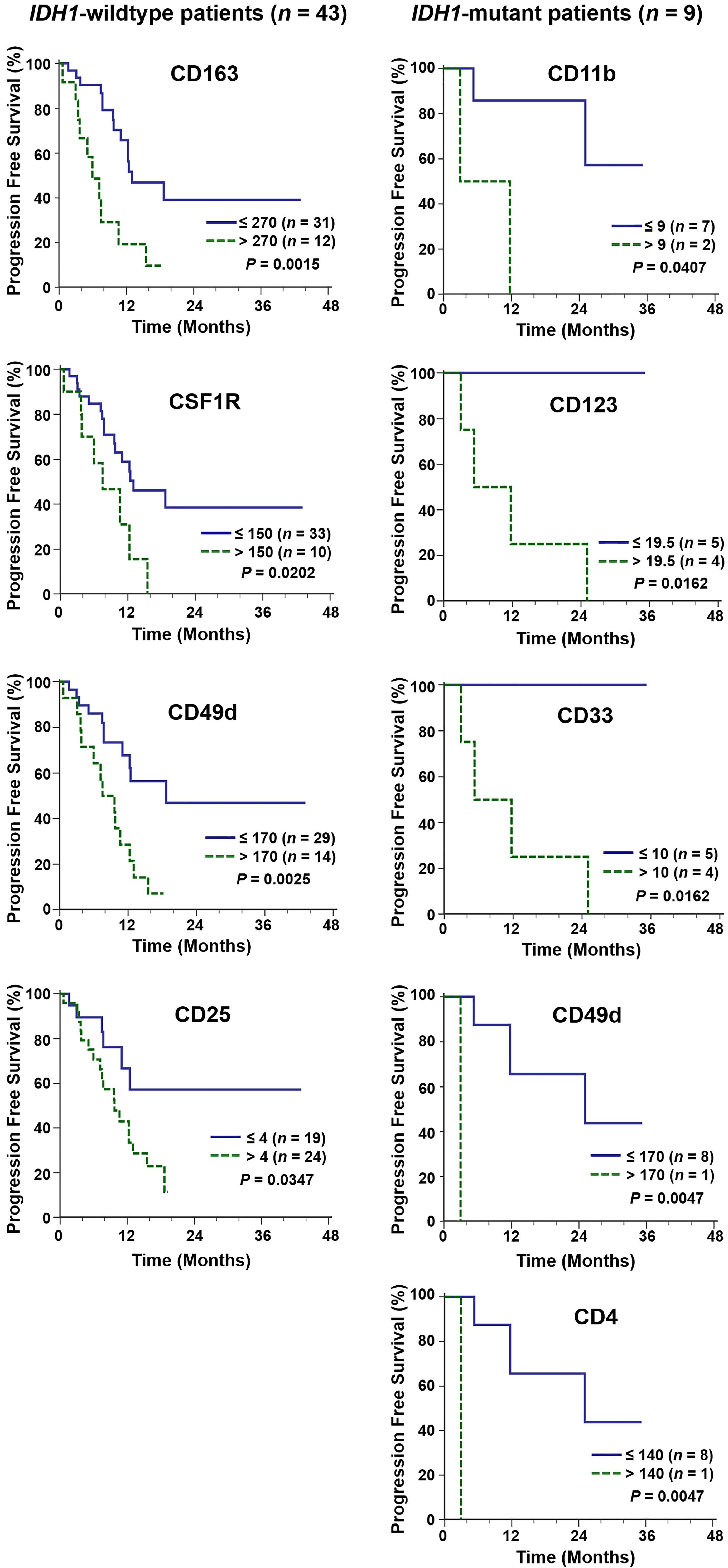


**Supplementary Figure 2. Kaplan-Meier estimates of PFS according to the expression level of immune cell markers and IDH1 mutation status.**

**Supplementary Tables**

**Table 1. Kaplan Meier analysis of PFS according to expression level of imm**une cell markers

|  |  | Median PFS (Month) | 95% CI | *P* |
| --- | --- | --- | --- | --- |
| CD49d | ≤170 (n = 37) | 25.1 | 12.3 - 25.1 | 0.0002 |
|  | >170 (n = 15) | 7.5 | 3.8 - 10.6 |  |
| CD11b | ≤9 (n = 31) | 25.1 | 11.0 - 25.1 | 0.0132 |
|  | >9 (n = 21) | 10.6 | 7.2 - 13.0 |  |
| CD123 | ≤19.5 (n = 24) | - | - | 0.0266 |
|  | >19.5 (n = 28) | 10.6 | 7.2 - 15.5 |  |
| CD33 | ≤10 (n = 20) | - | - | 0.0342 |
|  | >10 (n = 32) | 11.0 | 7.5 - 15.5 |  |
| CD163 | ≤270 (n = 39) | 13.0 | 11.7 - 18.7 | 0.0043 |
|  | >270 (n = 13) | 7.2 | 3.7 - 15.5 |  |
| CD25 | ≤4 (n = 25) | - | - | 0.0059 |
|  | >4 (n = 27) | 10.6 | 7.2 - 13.0 |  |
| CD63 | ≤116 (n = 26) | 25.1 | 11.0 - 25.1 | 0.0352 |
|  | >116 (n = 26) | 10.6 | 7.2 - 13.0 |  |
| CD8 | ≤16 (n = 26) | - | - | 0.0245 |
|  | >16 (n = 26) | 11.0 | 7.5 - 13.0 |  |

95% CI - 95% confidence interval

Table 2. Kaplan Meier analysis of PFS according to expression level of immune cell markers in IDH1-wildtype patients

|  |  | Median PFS (Month) | 95% CI | *P* |
| --- | --- | --- | --- | --- |
| CD163 | ≤270 (n = 31) | 13.0 | 11.0 - 18.7 | 0.0015 |
|  | >270 (n = 12) | 5.9 | 3.7 - 10.6 |  |
| CSF1R | ≤150 (n = 33) | 13.0 | 9.6 - 18.7 | 0.0202 |
|  | >150 (n = 10) | 7.5 | 3.8 - 12.3 |  |
| CD49d | ≤170 (n = 29) | 18.7 | 11.0 - 18.7 | 0.0025 |
|  | >170 (n = 14) | 7.5 | 3.8 - 12.3 |  |
| CD25 | ≤4 (n = 19) | - | - | 0.0347 |
|  | >4 (n = 24) | 9.7 | 7.2 - 13.0 |  |

95% CI - 95% confidence interval

Table 3. Kaplan Meier analysis of PFS according to expression level of immune cell markers in IDH1 mutant patients

|  |  | Median PFS (Month) | 95% CI | *P* |
| --- | --- | --- | --- | --- |
| CD11b | ≤9 (n = 7) | - | - | 0.0407 |
|  | >9 (n = 2) | 3.0 | 3.0 - 11.7 |  |
| CD123 | ≤19.5 (n = 5) | - | - | 0.0162 |
|  | >19.5 (n = 4) | 5.3 | 3.0 - 25.1 |  |
| CD33 | ≤10 (n = 5) | - | - | 0.0162 |
|  | >10 (n = 4) | 5.3 | 3.0 - 25.1 |  |
| CD49d | ≤170 (n = 8) | 25.1 | 11.7 - 25.1 | 0.0047 |
|  | >170 (n = 1) | 3.0 | - |  |
| CD4 | ≤140 (n = 8) | 25.1 | 11.7 - 25.1 | 0.0047 |
|  | >140 (n = 1) | 3.0 | - |  |

95% CI - 95% confidence interval
